# Supplementary material for: Attention-deficit/hyperactivity disorder and serial missed appointments in general practice
Source: PLOS Ment Health. 2024 Jul 8;1(2):e0000045. doi: 10.1371/journal.pmen.0000045 (PMC12798550; doi:10.1371/journal.pmen.0000045)
Supplement: Attachment — Submitted filename: Response to Reviewers Comments.docx [file pmen.0000045.s001.docx]

Response to Reviewers Comments: PMEN-D-24-00011

Reviewer #1: The authors provide a data availability statement explaining their access to the data and how this can be made available to others if required.

The manuscript focuses on a very topical and interesting issue. Offering useful insights on a challenging issue for general practice.

Many thanks.

Some observations and queries:

Missed appointments were defined based on recorded time logs for appointments using methods described in previous papers. (pg8)

Some further detail to explain the time logs would be useful here rather than the reference to other papers. It would be useful to know if missed appointments were defined any further, for example did this include cancelled appointments, appointments where children (or adults) may have been recorded as ‘not brought’ to appointments?

We agree and have added further information to the manuscript to clarify this point (rather than rely on information reported in previous papers). We can confirm that our analysis did not include cancelled appointments or ‘not brought’ appointments as these were not coded in the data provided.

Are the time logs linked to codes such as Did not attend or was not brought, as there are many potential options within systems to indicate a missed appointment.

Time codes (or a lack of) are used to derive Did not attend status. This has also been clarified in our revision.

• We observed that adults, compared to young people, are more likely to miss appointments irrespective of ADHD, (pg17) o It may have been useful to see some discussion here about difference in child and adult attendance at appointments, recognising that children are likely to be ‘brought’ to appointments by families/parents and therefore this may account for lower missed appointments. o

We agree and have added this as a limitation to our discussion. We can’t be sure if a child was brought vs attended on their own.

This relates to my earlier point re the explanation of time logs and if this considered where children or adults were not brought to appointments where systems have recorded this data.

• Children and adolescents with ADHD were in relative terms more likely than the adults in the sample to miss appointments.

o This point appears to contradict the earlier point re adults being more likely to miss appointments.

This has been corrected.

The conclusion offers a useful account of the implications of missed appointments. Have the authors considered the system implications for missed appointments. Inflexibility in healthcare systems, appointment availability, environmental considerations when attending appointments for people with ADHD may all be compounding factors in non-attendance and are worthy of some acknowledgement as these are issues explored in other populations such as learning disabilities.

Many thanks. We have considered this point but felt that an additional comment regarding this is somewhat overstepping what we can infer from our data.

This is an interesting area of study and a very useful piece of work to support and improve access to health services and address health inequalities.

Many thanks.

Reviewer #2: Great work! I was enjoying reading it! The work is great; you approached a very interesting and understudied field, connecting the mental health and behaviour of the patients. The study design is rigorous, and data analysis was conducted in accordance with all standards.

Many thanks

Reviewer #3: In my opinion, the manuscript is acceptable because it clearly and precisely explains the background of the study, the methods used, the results obtained, the conclusions, and the scope and limitations. They also include references to documents where the study procedures can be consulted in more detail. The authors state that the results have limited clinical implications, but this work is a pioneering study on this topic.

Many thanks.

Reviewer #4: PLOS Mental Health Title: Attention-Deficit/Hyperactivity Disorder and serial missed appointments in general practice Thank you for allowing me to review your manuscript, Attention-Deficit/Hyperactivity Disorder and serial missed appointments in general practice. This study examined missed appointments in General Practice (GP) and patients with ADHD. I am currently recommending a revise and resubmit, encouraging the authors to focus on shifting language to be less pathologizing (described below) and addressing some questions and limitations of the methods.

Overall: - This paper is grounded in the pathology paradigm, which is outdated and a direct opposition to the neurodiversity paradigm, which is embraced by many disabled people. I would consider revising the paper to remove some of the pathologizing language. The content is incredibly important, and as someone who identifies as ADHD, a very relevant and personal topic to me. However, language like “exposed individuals” and “not exposed” individuals sends the message that ADHD is something bad that can be transmitted through exposure.

We thank you for your bringing this to our attention and have revised the paper to remove pathologizing language. Our use of words like ‘exposed’ mirrors statistical language used to define treatment groups or controls (who, for example, may or may not have been exposed to a drug or virus). However, we completely agree that this is not appropriate in this instance. Our revision now aligns with a neurodiversity paradigm as suggested.

Introduction:

● No comments

Methods:

● Matched on sex and age o How is sex defined? Binary option? If that is all that is available, this should be listed as a limitation of the study, as many neurodivergent people identify as gender non-conforming.

This reflects a limitation of what is recorded in the medical records (sex is recorded but not gender). Interestingly, patients have for many years been able to declare their preferred gender to the GP, and the GP has to record it. It does involve a change in CHI number though, which makes it difficult to track patients who have transitioned in administrative records.

The impact of sex and gender was not something we ever planned to investigate, that these were not mentioned anywhere in the paper except insofar as we matched on sex as recorded by the GP. However, even if there been multiple options for gender we would have likely had to remove these from these our analysis due to issues associated with limited statistical power.

● Strong description of how sample was identified

Many thanks

● Description of methods in other papers o If there is no concern with space, I would recommend that methods described in previous papers are also included in this one. As a reader, I would like to be able to read this paper and understand the strengths and limitations of the methods without needing to move to another paper to review.

This aligns with comments from Reviewer 1 and we have extended these sections so readers no longer need to rely on another paper/papers.

Results & Discussion

● No comments
